# Buffalo State Insane Asylum and Clinical and Didactic Teaching in Medical Colleges upon the Subject of Insanity

**Published:** 1870-06

**Authors:** 


### Editorial Department.

### Buffalo State Insane Asylum and Clinical and Didactic Teaching in Medical Colleges upon the Subject of Insanity.

The Medical profession in this vicinity is already deeply interested in the progress of the Asylum for the Insane, about to be built by the State of New York in the City of Buffalo, and our readers will many of them, take much pleasure in learning of its prospects; we have therefore taken pains to inquire into the present standing of its affairs. The Board of Managers has been appointed by the Governor, and held its first meeting, organizing by the election of James P. White, of Buffalo, as President.

The following Committees have also been appointed:

*Committee on Plans.*—John P. Gray, M. D., James P. White, M. D., Dennis Bowen, Esq.

This Committee has made some progress, has examined several institutions, and is now engaged in the meeting of the Superintendents of Insane Asylums in Hartford, where they propose to examine the newly remodeled institution in that city, and afterwards to visit most of the New England institutions for the care of the insane.

A *plan* has been nearly matured and completed, which is thought to have better arrangement for ventilation and classification of patients than any ever yet adopted. In view of the practical wisdom and large experience of the committee, not only in constructing buildings for that purpose, but of the chairman, Dr. Gray, also, in the care and treatment of the insane, the friends of the enterprise may confidently expect that this institution will possess advantages nowhere equalled; that it will be a model for imitation in the erection of Insane Asylums, the world over.

The city is proceeding as rapidly as possible in obtaining title to the site, and the State has appropriated fifty thousand, which will be this year expended in grading, fencing, sewerage, laying foundation, &c., &c., &c. This is under the direction of the *Committee on Grounds,* Joseph Warren, Esq., A. P. Lanning, Esq., and George R. Yaw, Esq. A topographical survey of the grounds is being made, and thus the work will be conducted upon a well digested plan.

In the location of this Asylum in Buffalo, every right-minded lover of humanity will take pleasure, upon the general principle that it is here capable of doing the greatest good to the greatest number; but there is another reason why the profession may look with satisfaction upon this location of the Asylum. It is well known how active and earnest Prof. White has been in this enterprise, and that to him may be traced the conception and much of the progress thus far made, in which he has been most ably and efficiently aided by Senators A. P. Nichols and L. L. Lewis, and Joseph Warren, Esq., and others, to whom many thanks are due from the citizens of Western New York and vicinity. Prof. White, doubtless from the very first, saw that it would afford opportunity for clinical teaching of insanity in the University of Buffalo—his favorite institution, and thus aid in supplying a deficiency in the curriculum of medical education. Most of the profession have already noticed how actively and efficiently he has labored to introduce didactic and clinical instruction on insanity as a part of the course of medical education, and have been gratified by the favor with which his propositions have everywhere been received. It will be remembered that his first presentation of the subject was in his address before the New York State Medical Society, which resulted in the appointment of a committee to report at the next meeting. Again, in the American Medical Association his resolutions on this subject were unanimously adopted; and more recently at the present meeting of the Superintendents of Insane Asylums at Hartford, the same propositions are introduced by him and received with marked approbation. The idea is, that physicians are often called to treat insanity in its earliest stages, and therefore should be familiar with its nature and best modes of treatment, the same as with other diseases. This subject of clinical teaching in insanity, has been most ably presented to the profession and public by Dr. E. P. Gray, of Utica, in an article published in the *Journal of Insanity*, for April, 1870, from which we take great pleasure in quoting a paragraph which gives some of the facts in the recent history of this subject:

"In February, 1868, in the annual address, as President of the Medical Society of the State of New York, the editor of this journal,* advocated the absolute necessity of didactic and clinical teaching of insanity and other cerebral and nervous disorders, in our medical schools, and quoted the experience of Griesinger in verification of its thorough practicability. Within the past year Dr. Hammond, Professor of the Diseases of the Mind and Nervous System, and of Clinical Medicine, in the Bellevue Hospital Medical College of New York, has opened a clinic on nervous diseases and insanity, and Prof. J. Keating Bauduy, of St. Louis College of Physicians and Surgeons, has instituted a clinic at Saint Vincent Insane Asylum, St. Louis, Mo.

Professor James P. White, of Buffalo Medical College, in his inaugural address as President of the Medical Society of the State of New York, in February, 1870, thus urged this subject again on the attention of the profession: 'There is one step in a forward direction, which should now be taken, and which seems to me this Society can promote by its influence and indorsement. I allude to the teaching of Psychological Medicine, as a part of the curriculum of the college course. Is it less important that diseases of the mind, always involving the brain and nervous system, with their sympathetic connections and influences should be properly taught and illustrated with cases, than any of those maladies, now deemed essential to be clinically taught in any institution? The attention of the profession is beginning to receive this direction; some steps have already been taken towards its accomplishment, and I believe the medical mind is thoroughly ripe for action, if commended by this Society

**Journal of Insanity*—Dr. E. P. Gray, of Utica.

in suitable and proper terms. Permit me, therefore, to suggest the appointment of a select committee, to take this matter duly into consideration, and recommend to the Society what action, if any, it should take in its furtherance.'"

Drs. John P. Gray, of Utica, Joseph C. Hutchinson, of Brooklyn, and Charles H. Porter, of Albany, were appointed a committee on the President's address, and reported a resolution, which was unanimously adopted, expressing the necessity of making didactic teaching and clinical instruction in insanity and all other cerebral and nervous diseases, obligatory as a part of the curriculum of study in our medical colleges, and directing the Secretary of the Society to send a copy of this resolution to the faculty of each medical college of the State.

Thus this important subject has received the indorsement of one of the oldest, and probably the most influential of the medical societies in the country. It is greatly to be regretted that so large a majority of the institutions for the treatment of the insane are remote from established medical schools. However, a sufficient number are in proximity to medical colleges to inaugurate at once this important advance in medicine.

In the location of the new State Asylum, in the 8th judicial district of New York, the past year, the Commissioners of location, having this in view, and being all medical men, *gave unanimous preference to Buffalo over other localities presented. The established medical college in that city being in proximity to the ground presented for the asylum, the advantages of clinical instruction can be at once realized.

In conclusion, Dr. Gray says:

It is evident that while psychological medicine must still be a speciality, from the necessity of most cases of insanity requiring to be cared for and treated in hospitals, it can no longer be severed from general medicine. It is inseparable from medical science. Insanity is too intimately interrelated with all disordered conditions of the organism to isolate it in general study and practice. The demands of medical science and the indications outlined, point to a union. Medicine is one study; all departments are inseparably related by psychological laws and pathological conditions. Medicine is no longer an art, but a grand science; and while it is too vast for the comprehension of any mind, it is nevertheless a unit.

———: o :———

### Death of Sir James Y. Simpson, M. D., of Edinburgh. Resolutions adopted by a meeting of American Physicians at Washington.

A meeting of such of the physicians of the United States as were then present at Washington, was held in the Army Medical Museum, May 10th, of which Dr. Thomas Miller was elected President, immediately upon the organizing of

*Dr. John P. Gray, Superintendent State Asylum, Utica; Prof. James P. White, of Buffalo; Dr. Wm. B. Gould, of Lockport; Dr. Milan Baker, of Warsaw, and Dr. Thomas D. Strong, of Westfield.

